# Development and Testing of a Novel Microstrip Photocathode ICCD for Lunar Remote Raman Detection

**DOI:** 10.3390/s25051528

**Published:** 2025-02-28

**Authors:** Haiting Zhao, Xiangfeng Liu, Chao Chen, Weiming Xu, Jianan Xie, Zhenqiang Zhang, Ziqing Jiang, Xuesen Xu, Zhiping He, Rong Shu, Jianyu Wang

**Affiliations:** 1 Key Laboratory of Space Active Opto-Electronics Technology, Shanghai Institute of Technical Physics, Chinese Academy of Sciences (CAS), Shanghai 200083, China; zhaohaiting@mail.sitp.ac.cn (H.Z.); liuxiangfeng@mail.sitp.ac.cn (X.L.); xiejianan@sitp.ac.cn (J.X.); zhangzhenqiang@mail.sitp.ac.cn (Z.Z.); jzq2166@mail.sitp.ac.cn (Z.J.); hzping@mail.sitp.ac.cn (Z.H.); shurong@mail.sitp.ac.cn (R.S.); jywang@mail.sitp.ac.cn (J.W.); 2 University of Chinese Academy of Sciences, Beijing 100049, China; 3 Northern Night Vision Technology Co., Ltd., Kunming 650217, China; cc-star@163.com; 4 School of Physics and Optoelectronic Engineering, Hangzhou Institute for Advanced Study, University of Chinese Academy of Sciences, Hangzhou 310024, China; xuxuesen@ucas.ac.cn

**Keywords:** lunar exploration, Raman spectroscopy, image intensifier, ICCD, time gating

## Abstract

The intensified charge-coupled device (ICCD), known for its exceptional low-light detection performance and time-gating capability, has been widely applied in remote Raman spectroscopy systems. However, existing ICCDs face significant challenges in meeting the comprehensive requirements of high gating speed, high sensitivity, high resolution, miniaturization, and adaptability to extreme environments for the upcoming lunar remote Raman spectroscopy missions. To address these challenges, this study developed a microstrip photocathode (MP-ICCD) specifically designed for lunar remote Raman spectroscopy. A comprehensive testing method was also proposed to evaluate critical performance parameters, including optical gating width, optimal gain voltage, and relative resolution. The MP-ICCD was integrated into a prototype remote Raman spectrometer equipped with a 40 mm aperture telescope and tested under outdoor sunlight conditions. The experimental results demonstrated that the developed MP-ICCD successfully achieved a minimum optical gating width of 6.0 ns and an optimal gain voltage of 870 V, with resolution meeting the requirements for Raman spectroscopy detection. Under outdoor solar illumination, the prototype remote Raman spectrometer utilizing the MP-ICCD accurately detected the Raman spectra of typical lunar minerals, including quartz, olivine, pyroxene, and plagioclase, at a distance of 1.5 m. This study provides essential technical support and experimental validation for the application of MP-ICCD in lunar Raman spectroscopy missions.

## 1. Introduction

Raman spectroscopy is a technique that generates a characteristic spectrum as a result of inelastic scattering, which occurs when photons interact with matter [1]. It is a highly versatile tool that is employed in a wide range of fields, including mineral analysis, chemical analysis, and life sciences [2,3,4]. One of the key advantages of Raman spectroscopy is that it provides sharp and non-overlapping characteristic peaks. Furthermore, the non-contact, remote, and non-destructive nature of the analysis, in addition to the lack of need for sample preparation, makes it a highly suitable method for mineral analysis of terrestrial and extraterrestrial samples [5]. In particular, it performs a pivotal function in the scientific exploration of near-Earth celestial bodies, including the Moon and Mars.

In the planned Chinese Chang’e-7 mission, scheduled for launch in 2026, the rover will be equipped with a lightweight and compact remote Raman spectrometer to analyze the mineral composition of the lunar south pole surface and to search for potential water ice [6,7]. The objective of this instrument is to provide direct evidence for understanding the Moon’s geological and chemical environment. However, remote Raman spectroscopy on the Moon faces multiple challenges, including the inherently weak Raman scattering signals, susceptibility to interference from fluorescence, and solar background radiation [8,9,10]. The lunar rover requires sufficient sunlight to provide solar energy, and Raman spectroscopy detection activities are primarily conducted under sunlight to ensure adequate energy supply and the safe operation of the equipment. Water ice may exist near the lunar south pole. The Raman scattering efficiency of water ice is relatively low, and its content may be very limited. Similar to minerals, detecting water ice requires a highly sensitive Raman spectroscopy detector. Furthermore, stringent engineering constraints on system size, weight, and power demand a compact and lightweight design, while still achieving high performance. Ensuring a high signal-to-noise ratio (SNR) and accurate scientific data poses significant challenges to both the system design and the selection of key components.

Time-gated technology, by precisely controlling temporal resolution, enables detectors to capture transient signals within specific time windows, effectively suppressing background light and fluorescence interference [11,12]. This is a critical technique for achieving high SNR measurements of weak remote Raman signals. Current major time-gating implementations include the optical Kerr gate (Kerr-gate) based on the Kerr effect, time-gated single-photon avalanche diodes (TG-SPADs), and the intensified charge-coupled device (ICCD). The Kerr-gate operates by inducing a nonlinear refractive index change in a Kerr medium using a strong laser pulse, achieving picosecond-level temporal resolution [13]. However, its reliance on high-pulse-energy lasers and intricate optical systems restricts its practical applicability. The TG-SPAD achieves gating by controlling the bias voltage, offering a temporal resolution ranging from tens to hundreds of picoseconds [14,15]. Nevertheless, further technological refinement is required to address the challenges posed by the stringent reliability and stability demands of lunar exploration.

The ICCD integrates an image intensifier with a CCD device, enabling gating through the modulation of the photocathode voltage [16]. This configuration facilitates gating widths at the nanosecond level, along with notable advantages such as high sensitivity and a compact design. For instance, the SuperCam remote Raman spectrometer, which was integrated for NASA’s 2020 Mars Exploration Mission, employs ICCD technology with a minimum gating width of 100 ns and a 110 mm-aperture telescope [17,18]. This sophisticated instrument is capable of efficiently detecting Raman spectral signals of carbonates, sulfates and organics in the range of 2–7 m, thereby providing significant support for the characterization of the Jezero Crater region and the optimization of sample selection [19].

Early incarnations of the ICCD were plagued by two significant issues: firstly, they exhibited elevated levels of noise in their functioning due to the nascent state of the technologies concerned with both the image intensifier and the detector; and secondly, they demonstrated limited capacity for dynamic range, once again a consequence of the immaturity of the technologies employed [20]. However, advancements in materials science, electronics, and optical device technologies have led to significant performance breakthroughs in modern ICCDs. These include enhanced temporal resolution, higher gain, increased sensitivity, and reduced noise characteristics. These advancements have facilitated the widespread application of ICCDs in fields such as Raman spectroscopy, time-resolved fluorescence imaging, and plasma diagnostics [16,21,22]. In the context of remote lunar Raman spectroscopy, key performance metrics for ICCDs include switching speed, sensitivity, and resolution, all of which critically influence detection outcomes [23,24]. Additionally, miniaturization is a vital consideration to meet the stringent engineering constraints of lunar exploration missions.

Based on the structural characteristics of ICCDs, we conducted an in-depth analysis of their primary components and designed an ICCD tailored to the specific requirements of lunar Raman spectroscopy, aiming to achieve optimal detection performance. The switching speed of ICCD is primarily influenced by the photocathode material and structure of the image intensifier [25]. A photocathode with low surface sheet resistance and minimal equivalent capacitance is essential [26,27,28]. To meet these requirements, we selected an S25(Na_2_KSb) photocathode and coated its surface with a thin metallic conductive layer to reduce surface current transmission time. Additionally, a 25 × 3 mm microstrip photocathode design was adopted, effectively lowering the equivalent capacitance between the photocathode and the microchannel plate (MCP), thereby achieving nanosecond-level gating speed. This design was designated as the MP-ICCD. To improve sensitivity, we added a reflection-reducing coating between the optical input window and the photocathode. Furthermore, an integrated design combining the phosphor screen and fiber optic taper was employed, simplifying the coupling process. This approach not only improved resolution but also contributed to the miniaturization and high integration of the device.

To evaluate the key characteristics of the developed MP-ICCD, we proposed a comprehensive testing methodology encompassing critical performance metrics such as optical gating width, optimal gain voltage, relative resolution, and Raman signal detection. Using a prototype remote Raman spectrometer equipped with the MP-ICCD, we successfully detected Raman spectra of typical lunar minerals—including quartz, olivine, orthopyroxene, and plagioclase—at a distance of 1.5 m under outdoor sunlight conditions.

The remainder of this paper is structured as follows: Section 2 introduces the composition and working principle of the MP-ICCD and outlines the validation methods for its comprehensive performance. Section 3 presents the test results for optical gating width, optimal gain voltage, and relative resolution, along with Raman signal detection under outdoor sunlight background. Section 4 provides a detailed discussion of the test results. Finally, Section 5 summarizes the main findings and highlights the potential applications for future research.

## 2. Materials and Methods

### 2.1. Development of MP-ICCD

#### 2.1.1. Composition and Working Principle of MP-ICCD

**Figure 1:**
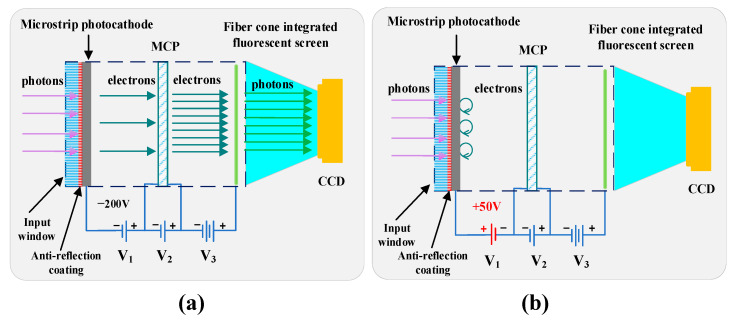
Schematic diagram of MP-ICCD composition and working principle. (**a**) MP-ICCD “on” state. (**b**) MP-ICCD “off” state.

Figure 1 illustrates the structural composition and working principle of the developed MP-ICCD. The device primarily consists of an image intensifier optically coupled to a CCD. The image intensifier is composed of an input window with an anti-reflection coating, a microstrip photocathode, an MCP, and an integrated phosphor screen with a fiber optic taper. Incoming light passes through the input window and reaches the photocathode, where it is converted into electrons.

These electrons are directed into the MCP, where they undergo multiple stages of multiplication and acceleration, achieving high gain. The amplified electrons are then accelerated toward the phosphor screen, where they are converted back into photons. These photons are focused onto the CCD via the fiber optic taper. Within the CCD, the incident photons are converted into electrical charges by the photosensitive pixels. These charges are then transferred and amplified, generating voltage signals. Finally, these voltage signals are digitized into spectral data using an analog-to-digital converter.

The gating functionality of the MP-ICCD is achieved by adjusting the voltage *V*_1_ between the photocathode and the MCP within the image intensifier. As shown in Figure 1a, when *V*_1_ is set to −200 V, a forward electric field is established, allowing photoelectrons to be emitted from the photocathode and enter the MCP for multiplication and amplification. In this state, the image intensifier is “on”. In contrast, as illustrated in Figure 1b, when *V*_1_ is adjusted to +50 V, the electric field reverses direction, preventing photoelectrons from reaching the MCP and subsequent stages. In this state, the image intensifier is “off”. By rapidly switching the amplitude and polarity of *V*_1_, the MP-ICCD achieves fast gating functionality, enabling precise temporal control of signal acquisition. The voltage *V*_2_ controls the gain by adjusting the voltage across the two ends of the MCP, with a range of 0–1000 V. The voltage *V*_3_ is fixed at 5800 V to accelerate the multiplied electron stream, enabling it to energetically strike the phosphor screen and produce an output signal with enhanced brightness. During operation, the total power consumption of the MP-ICCD system, including the detector, driver, and high-voltage power supply, is approximately 5.9 W.

#### 2.1.2. Development of Image Intensifier

The developed image intensifier is an improved version based on super second-generation technology, featuring the following key characteristics: the use of an input window with an anti-reflection coating, a microstrip photocathode, and an integrated phosphor screen with a fiber optic taper. After a comprehensive evaluation of reliability, availability, and performance stability, we selected the S25 photocathode, which exhibits a spectral response range of 380–900 nm and achieves a quantum efficiency of approximately 12–16% within the target spectral range of 532–785 nm. These innovations are designed to achieve high sensitivity, fast gating speed, high resolution, and compact size. The following sections provide a detailed discussion of these critical features.

1. When signal light enters the image intensifier, it first passes through the input window, where the optical signal is transmitted through its inner surface to the photocathode for photoelectric conversion. However, during this process, some light is reflected by the photocathode, reducing the efficiency of photon-to-electron conversion and subsequently affecting the sensitivity of the photocathode. To enhance sensitivity, a reflection-reducing coating was applied to the inner surface of the input window prior to the fabrication of the photocathode. This process significantly improved the photocathode’s absorption rate for incident light, thereby effectively increasing the photoelectric conversion efficiency;
2. The gating speed of the image intensifier is primarily determined by the equivalent resistance and transient equivalent capacitance of the photocathode. To reduce the equivalent resistance, methods such as depositing a conductive layer on the photocathode or doping are commonly employed. However, these approaches often compromise sensitivity. By designing a microstrip photocathode structure, the equivalent capacitance can be significantly reduced, effectively improving the gating speed without sacrificing sensitivity. The feasibility of this approach has been theoretically analyzed and experimentally validated by Mei Zhang et al. [29].

**Figure 2:**
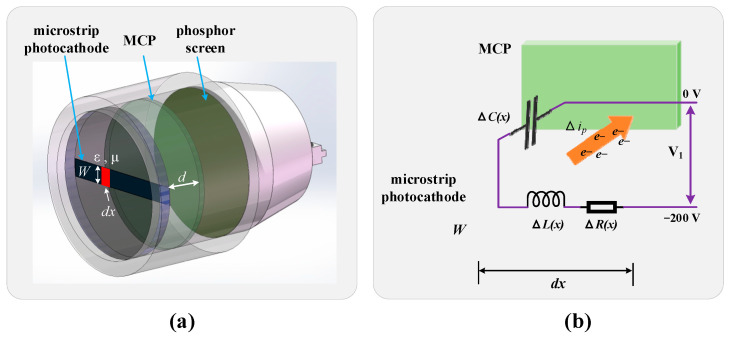
(**a**) Model of microstrip photocathode image intensifier. (**b**) Equivalent distributed circuit of photocathode.

Figure 2a,b illustrate the model and equivalent circuit of the microstrip photocathode, respectively. In this model, *W*, *ϵ*, and *μ* represent the width of the microstrip photocathode, the dielectric constant, and the magnetic permeability, respectively. *dx* denotes the distance over which the cathode voltage is transmitted during an infinitesimal time interval, and *d* is the distance between the microstrip photocathode and the MCP input surface. The parameters Δ*R(x)*, Δ*L(x)*, Δ*C(x)*, and Δ*i_p_* represent the transient equivalent resistance, inductance, capacitance, and the surface current induced by incident light over the differential length of the microstrip during the infinitesimal time interval. Specifically, the transient equivalent capacitance is given by Δ*C(x)* = *ϵ*⋅*W*⋅*dx*/*d*. The voltage *V(x,t)* on the microstrip photocathode characterizes the gating operation. Mei Zhang et al. [29] derived the differential equation for the microstrip cathode voltage, which is presented in Equation (1). This equation serves as the theoretical basis for understanding and optimizing the gating dynamics of the microstrip photocathode. The equation is as follows:

(1) $$\frac{\partial^{2}V(x,t)}{\partial x^{2}}=\mu \varepsilon \frac{\partial^{2}V(x,t)}{\partial t^{2}}+\frac{R_{s}\varepsilon W^{2}}{d}*\frac{\partial V(x,t)}{\partial t}+R_{s}W^{2}i_{p}\left(t\right)+\mu d\frac{\partial i_{p}\left(t\right)}{\partial t}.$$

**Figure 3:**
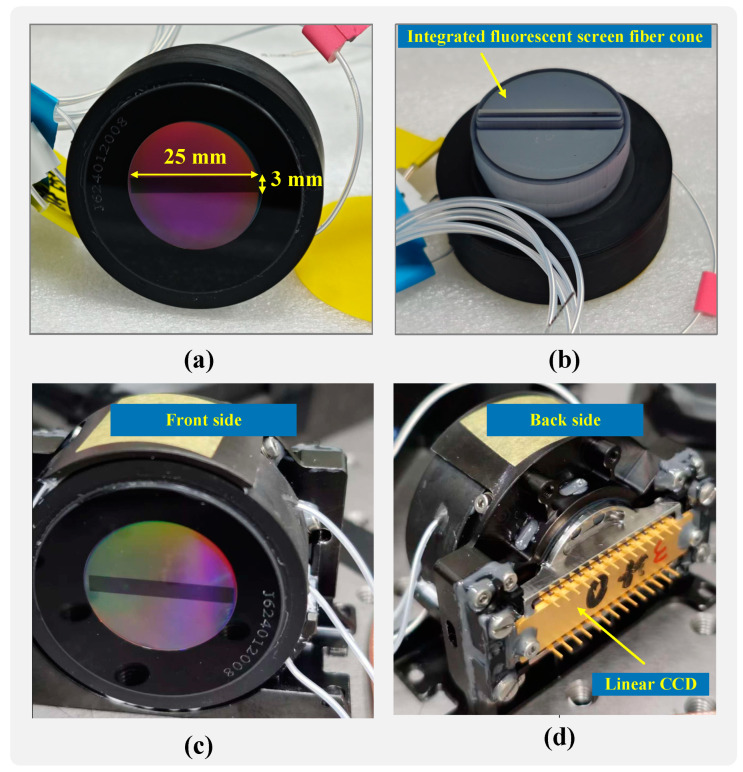
(**a**) Physical object of microstrip photocathode image intensifier. (**b**) Physical object of integrated fluorescent screen fiber cone. (**c**) Front side of the coupled MP-ICCD. (**d**) Backside of the coupled MP-ICCD.

To reduce Δ*C(x)*, the width *W* of the microstrip photocathode can be decreased. Since the objective is to acquire Raman spectra rather than imaging, *W* can be further minimized. However, if *d* (the distance between the microstrip photocathode and the MCP input surface) is too small, it may compromise the mechanical properties of the image intensifier. Given the requirements for lunar remote Raman spectroscopy, which necessitate strong resistance to impact and vibration, the design must balance these factors. Taking all considerations into account, *W* was ultimately set to 3.0 mm, and *d* was set to 0.12 mm. These design parameters are illustrated in Figure 3. This configuration ensures optimal gating performance while maintaining the mechanical robustness needed for lunar applications.

3. The phosphor screen of the image intensifier typically uses glass or fiber optic panels as the substrate and is fabricated using processes such as powder brushing, screen coating, and aluminum coating [30]. Its primary function is to convert the multiplied electrons generated by the MCP into visible light. The performance of the phosphor screen significantly impacts the resolution of the image intensifier [31]. Since Raman spectroscopy demands both high resolution and sensitivity, we optimized the phosphor screen in these two aspects. We selected P43(Gd_2_O_2_S:Tb) phosphor due to its high electro-optical efficiency, with an electron-to-photon conversion efficiency of approximately 20 photons per electron per kilovolt. The phosphor has a peak emission wavelength of 544.1 nm, a fluorescence lifetime of 0.6 ms, and an afterglow time of 1.2 ms. As shown in Figure 3b, we adopted an integrated fabrication process where one end of the fiber optic taper directly serves as the phosphor screen. This design not only simplifies the coupling process but also improves light transmission efficiency, contributing to enhanced resolution and sensitivity.

In addition to the components mentioned above, another critical part of the image intensifier is the MCP, which primarily functions to multiply the electrons emitted from the photocathode, thereby amplifying the signal. For lunar remote Raman spectroscopy, the MCP must meet stringent performance requirements, including high gain, low noise, and high resolution. The design parameters of the MCP include a channel pore diameter of 6 μm, a length-to-diameter ratio of 50:1, an open area ratio of ≥60%, and a dark current of ≤0.5 pA. After fabrication, all components are vacuum-sealed to assemble an image intensifier specifically tailored for lunar Raman spectroscopy applications. Preliminary performance tests were conducted on the developed image intensifier following standard testing protocols. The results demonstrated a sensitivity of 963 μA/lm, a brightness gain of 9500 cd/m^2^·lx, and an optical resolution of 57 lp/mm, meeting the requirements for high-performance lunar spectroscopy.

#### 2.1.3. Selection of the CCD Sensor

The selection of the sensor requires a comprehensive consideration of factors such as spectral range, quantum efficiency, noise level, and pixel resolution. Compared to conventional CMOS and sCMOS sensors, CCD sensors offer higher quantum efficiency and lower noise levels. Additionally, the pixel size of the CCD must be matched to the core diameter of the fiber optic taper to avoid excessively small pixel sizes that could compromise performance [32].

We selected a back-illuminated linear CCD sensor for this application. The sensor features an effective pixel array of 2048 × 1 with pixel dimensions of 14 × 1000 μm. At the peak emission wavelength of the P43 phosphor (544.1 nm), the sensor achieves a quantum efficiency exceeding 75%, ensuring high sensitivity and compatibility with the integrated system.

#### 2.1.4. Coupling Technology

In the coupling of the image intensifier and the CCD, key considerations include optical energy utilization, resolution loss, system integration, and reliability [33]. The goal is to ensure that the light signal emitted by the phosphor screen is efficiently transmitted to the CCD’s photosensitive surface, minimizing optical energy loss and avoiding signal distortion.

To achieve this, an integrated design was adopted, combining the phosphor screen and the fiber optic taper. This design requires only a single coupling step, simplifying the system and improving alignment. During the coupling process, precision mechanical positioning was employed to achieve micron-level accuracy. This approach minimizes resolution loss while ensuring structural integrity, resulting in enhanced mechanical performance and reliability.

### 2.2. Performance Evaluation Method

**Figure 4:**
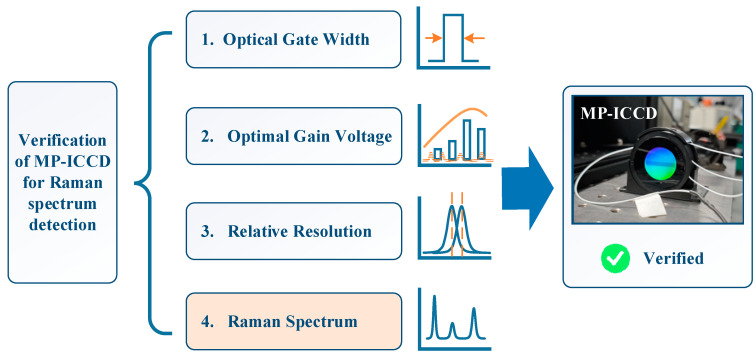
Performance verification scheme for MP-ICCD.

To optimize the development of the MP-ICCD and ensure its suitability for Raman spectroscopy applications, we proposed the performance validation scheme shown in Figure 4. This scheme includes four critical testing tasks: minimum optical gating width (MOGW) testing, optimal gain voltage testing, relative resolution testing, and Raman signal detection.

- MOGW and optimal gain voltage testing are conducted before the coupling process of the MP-ICCD components to focus on intrinsic device characteristics;
- Relative resolution testing is performed after the coupling process to evaluate the combined performance of the integrated system;
- Raman signal detection is validated by integrating the MP-ICCD into a prototype remote Raman spectrometer.

Each of these tests is designed to address specific performance aspects of the MP-ICCD and ensure its functionality aligns with the requirements of remote Raman spectroscopy. The following sections will provide detailed descriptions of the testing methodologies.

#### 2.2.1. Minimum Optical Gate Width Test

The MOGW is defined as the shortest time required for the image intensifier to fully switch between “on” and “off” states. It serves as a key metric for evaluating the gating speed of the MP-ICCD. The MOGW testing is conducted prior to the coupling of the MP-ICCD components to focus solely on the performance of the image intensifier.

**Figure 5:**
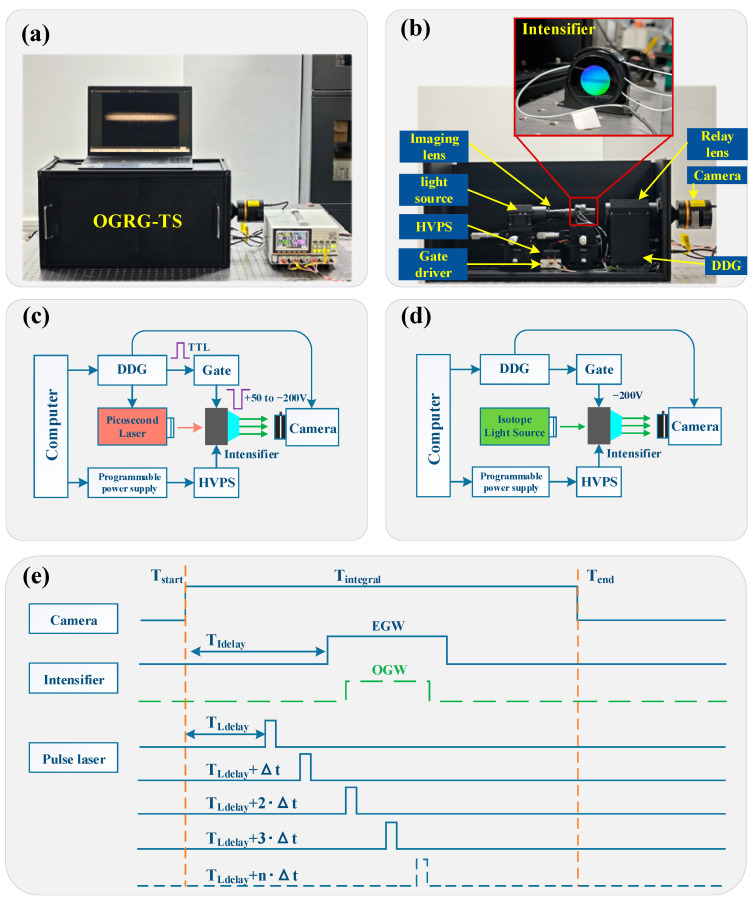
Test system and principles for OGRG-TS. (**a**) The physical setup of the test system; (**b**) the internal components of the test system; (**c**) the principle of minimum optical gate width testing; (**d**) the principle of optimal gain voltage testing; (**e**) timing diagram of optical gate testing. HVPS: high-voltage power supply, DDG: digital delay generator, EGW: electronic gate width, OGW: optical gate width.

The signal width emitted by the gating driver is referred to as the electronic gating width (EGW), while the effective time during which the image intensifier amplifies signal light is defined as the optical gating width (OGW). Due to the inherent delay in the photocathode’s response to gating signals, EGW is typically longer than OGW. Depending on the photocathode material and fabrication process, MOGW generally ranges from several hundred picoseconds to several hundred nanoseconds. The experimental setup, referred to as the Image Intensifier Optical Gate Width and Relative Gain Test System (OGRG-TS), is shown in Figure 5a. Figure 5b,c illustrate the internal structure of OGRG-TS and the principles of MOGW testing. The main components are described below:

- Light source: A pulsed laser (Photek Ltd., East Sussex, United Kingdom, LPG-405) with a pulse width of 100 ps and energy of 20 pJ is used as the light source. The central wavelength of this laser is 405 nm, which falls within the effective spectral response range of the photocathode. The system is equipped with replaceable light source modules for flexibility. A diffuser and a neutral-density filter are installed at the laser output to ensure the output intensity meets the testing requirements;
- Optical path: The optical path includes lenses to image the light source onto the image intensifier, a relay lens (Cis-systems Ltd., Xi’an, China, 1:1 Relay) for secondary imaging, and a monochrome camera (player-one Ltd., Suzhou, China, Apollo-M MAX PRO, resolution: 1920 × 1080) to capture the output signal;
- Control system: A digital delay generator (DDG, Cis-systems Ltd., D410) provides synchronized control, while a gating driver (Photek Ltd., GM300-3) generates the gating signals for the image intensifier;
- Power supply: The power system comprises a dual-channel high-voltage supply (adjustable MCP channel: 0–1000 V, fixed phosphor screen channel: 5800 V) and a programmable power supply (Gwinstek Ltd., Suzhou, China, GPP-4323). For testing, the MCP gain voltage is fixed at 800 V.

All components, except the programmable power supply, are enclosed in a light-tight dark chamber with dimensions of 60 × 30 × 30 cm. Closing the chamber door effectively blocks stray light, ensuring the accuracy and stability of the measurements. This system provides precise control and reliable measurements of MOGW, enabling a detailed characterization of the gating performance of the MP-ICCD. The high degree of integration and isolation ensures that external factors do not influence the results.

Figure 5e illustrates the control timing sequence for MOGW testing. The camera begins integration at time *T_start_*, and after a delay of *T_Idelay_*, the laser emits a light pulse. Following another *T_Idelay_*, the gating driver sends a gating signal to the image intensifier with a duration defined as the EGW, initially set to 3 ns. To ensure the complete measurement of the image intensifier’s opening and closing processes, the condition *T_Ldelay_* must be satisfied. At the end of the camera integration period, *T_end_*, the phosphor screen output images are saved and analyzed. Subsequently, while keeping all other parameters constant, the laser’s delay time *T_Ldelay_* is incrementally increased in steps of Δ*t* = 0.5 ns. After each adjustment, the brightness image of the phosphor screen output is captured until the laser pulse’s delay fully covers the EGW. Following this, the EGW is incrementally increased in 1 ns steps, and the testing process is repeated until the OGW is fully characterized. All relative timing relationships are configured using the digital delay generator (DDG), with full consideration of the insertion delays of each module.

#### 2.2.2. Optimal Gain Voltage Test

**Figure 6:**
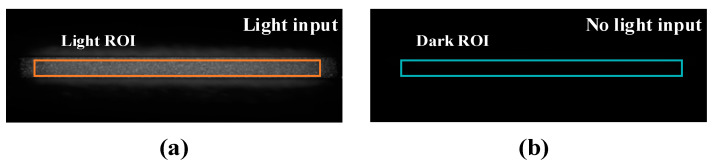
(**a**) ROI region with light input. (**b**) ROI region with no light input.

During the operation of the image intensifier, increasing the gain voltage enhances signal amplification but simultaneously raises the inherent noise level. Therefore, it is necessary to determine the gain voltage at which the SNR is maximized, referred to as the optimal gain voltage. This test is conducted prior to MP-ICCD coupling, and the determined optimal gain voltage is used as the preferred setting for Raman spectroscopy detection. Figure 5d illustrates the testing scheme for optimal gain voltage. The test is performed on the OGRG-TS platform, with the pulsed laser replaced by an isotopic light source (Cis-systems Ltd., IL-GREEN) capable of stable weak light emission. The neutral density filter at the light source output is adjusted to control the illuminance to the 10^−4^ lx level. A light-blocking shutter is installed at the light source output, which, when closed, allows measurement of the dark background of the image intensifier, with the mean dark background value representing the inherent noise of the image intensifier. During the test, the image intensifier operates in a constant-on mode, and the gain is varied by adjusting the voltage of the MCP channel on the high-voltage power supply. The gain voltage range is set from 600 to 950 V with a step size of 10 V. At each gain voltage, the phosphor screen brightness is captured by the camera under both light-source-on and light-source-off conditions. Figure 6a,b display the ROI regions under illuminated and non-illuminated conditions, respectively. The SNR at different gain voltages, *SNR(v)*, is calculated using Equation (2), and the gain voltage corresponding to the maximum *SNR(v)* is identified as the optimal gain voltage, *V_optimal_gain___v_*. The formula for SNR calculation is as follows:

(2) $$\mathrm{SNR}\left(v\right)=\frac{\sum_{i-1}^{N_{\mathrm{light}}}P_{\mathrm{light},v,i}}{N_{\mathrm{light}}}/\frac{\sum_{i-1}^{N_{\mathrm{dark}}}P_{\mathrm{dark},v,i}}{N_{\mathrm{dark}}}$$

here, *v* represents the gain voltage; *P_light,v,i_* and *P_dark,v,i_* denote the pixel values within the ROI of the illuminated and dark regions, respectively, at gain voltage *v*; and *N_light_* and *N_dark_* represent the total number of pixels in the illuminated and dark region ROIs, respectively.

#### 2.2.3. Relative Resolution Test

**Figure 7:**
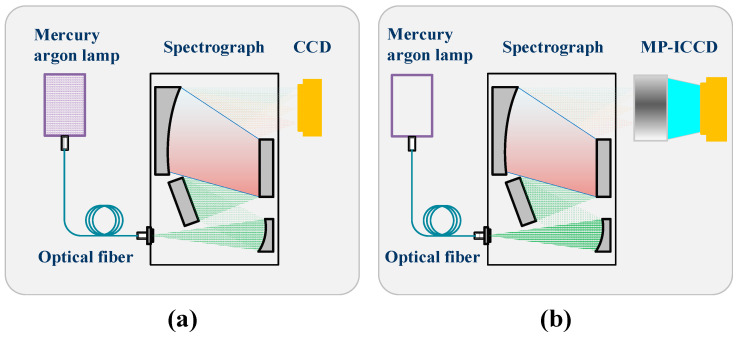
(**a**) CCD spectral resolution measurement. (**b**) MP-ICCD spectral resolution measurement.

The relative resolution test is designed to evaluate the resolution degradation of the MP-ICCD after coupling. By comparing the resolution of the coupled MP-ICCD with that of the uncoupled CCD, the resolution loss of the MP-ICCD can be quantified. Figure 7a illustrates the CCD resolution testing setup, which utilizes a mercury–argon lamp (WYOPTICS Ltd., Shanghai, China, HG-1) as the light source. This lamp emits atomic spectral lines with picometer-level spectral width. The light source is transmitted through an optical fiber to a spectrometer with a slit width of 25 μm and a spectral range of 532–785 nm. The spectrometer disperses the incident light into beams of different wavelengths and focuses them onto the CCD detector for signal acquisition. The resolution of the CCD is determined by the full-width at half-maximum (FWHM) of the spectral lines recorded on the CCD, expressed in terms of pixel count.

Figure 7b illustrates the resolution testing setup for the MP-ICCD, which is similar to the setup shown in Figure 7a, with the only difference being that the detector is replaced with the MP-ICCD. The resolution is defined as the FWHM of the spectral lines recorded on the MP-ICCD, expressed in terms of pixel count. All tests are conducted in a dark environment to eliminate interference from ambient light.

#### 2.2.4. Raman Spectroscopy Detection

**Figure 8:**
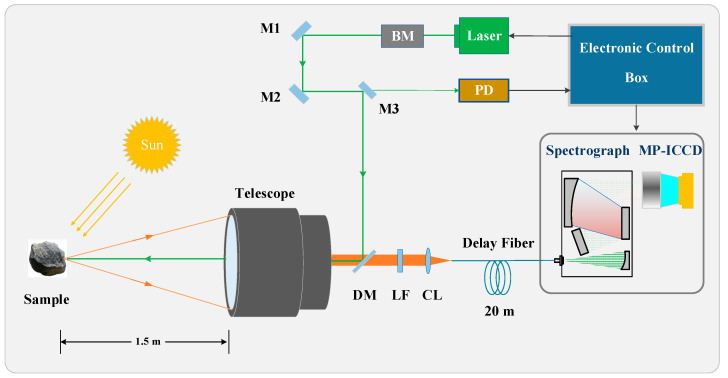
Schematic diagram of TG-LRS. BM: beam expander, PD: photodetector, DM: dichroic mirror, LF: long-pass filter, CL: collection lens.

To preliminarily validate the performance of the developed MP-ICCD in Raman spectroscopy detection, it was installed in a time-gated lunar remote Raman spectroscopy system (TG-LRS) for Raman spectrum acquisition testing. The testing principle is illustrated in Figure 8. The TG-LRS system primarily consists of the following components: a compact transmissive telescope (aperture: 40 mm), a passive Q-switched pulsed laser (center wavelength: 532 nm, pulse width: 1 ns, repetition rate: 1 kHz, energy: 100 µJ), a beam expander (BM), laser mirrors (M1, M2, M3), a photodetector (PD), a dichroic mirror (DM), a long-pass filter (LF), a collection lens (CL), and a spectrometer. We have selected a delay fiber length of 20 m and a core diameter of 50 µm. This length ensures that the delay from the PD receiving the emitted laser pulse to the opening of the MP-ICCD gate is less than the sum of the path delay and the fiber delay. Additionally, we have fully considered a margin to ensure that the chosen delay fiber length can accommodate Raman spectral detection requirements across all possible detection distances. Signal acquisition is controlled by an electronic control box. The spectrometer used in this test is the same as the one employed in the relative resolution tests.

The test was conducted under outdoor sunlight conditions, with the measured sunlight illuminance on the sample surface being 43,700 lx. The sample was placed 1.5 m away from the telescope. The pulsed laser emitted by the laser source was directed via M1, M2, M3, and DM, then focused onto the sample surface by the telescope. The Raman signal excited from the sample was collected by the telescope and passed through DM and LF before being coupled into the delay fiber by CL. After being delayed by the optical fiber, the signal reached the spectrometer, where it was dispersed and detected by the MP-ICCD.

Before testing, spectral calibration was performed using a mercury–argon lamp. During the test, the parameters for Raman spectroscopy acquisition were adjusted based on the results from the MOGW test and the optimal gain voltage test to ensure optimal system performance.

## 3. Results

### 3.1. Minimum Optical Gate Width

During the test, the EGW started at 3 ns and was incrementally increased by 1 ns at each step. For each EGW value, the laser pulse delay time *T_Ldelay_* was adjusted starting from 0 ns in increments of 0.5 ns. After each adjustment of *T_Ldelay_*, the brightness of the phosphor screen output was analyzed.

**Figure 9:**
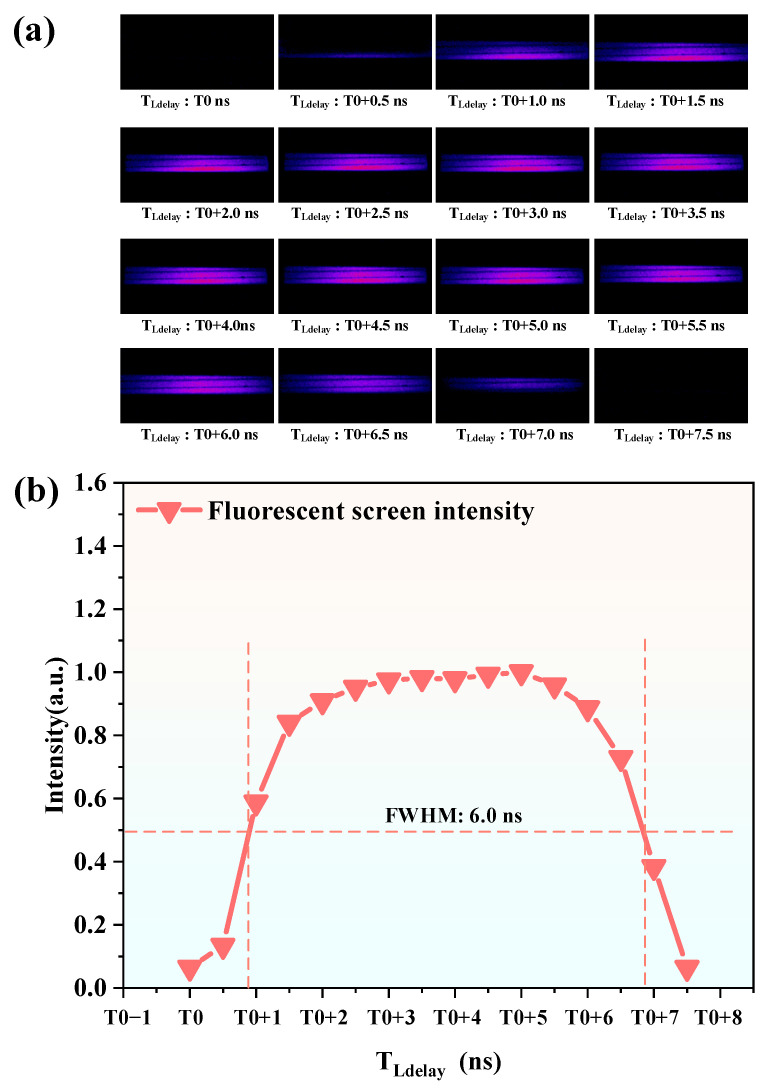
(**a**) Pseudo-color pictures of the entire process of opening and closing the intensifier of MP-ICCD. (**b**) Minimum optical gate width.

Through testing, the complete opening and closing process of the image intensifier was observed when EGW reached 12 ns. Figure 9a illustrates the corresponding test results, showing the brightness variation of the phosphor screen as a function of the laser pulse delay time *T_Ldelay_* from *T*_0_ to *T*_0_ + 7.5 ns, represented in pseudo-color. Here, *T*_0_ is the delay time *T_Ldelay_* at which phosphor screen brightness first begins to be detected.

Figure 9b further illustrates the relationship between the phosphor screen brightness and *T_Ldelay_*. The brightness of the phosphor screen is represented by the sum of pixel values in the central bright region of each image, normalized for clarity. It can be observed that at smaller *T_Ldelay_* values (e.g., *T*_0_ and *T*_0_ + 0.5 ns), the phosphor screen shows almost no signal, indicating that the gating mechanism was not fully open when the laser pulse reached the image intensifier. As *T_Ldelay_* increases, the phosphor screen brightness gradually intensifies, reaching its peak between *T*_0_ + 3.0 ns and *T*_0_ + 5.0 ns. During this 2 ns interval, the brightness remains almost constant, indicating that the gating mechanism was fully open when the laser pulse arrived. Beyond *T*_0_ + 5.0 ns, the signal starts to weaken and eventually diminishes almost completely at *T*_0_ + 7.5 ns, signifying that the gating mechanism had already closed by the time the laser pulse arrived.

The effective gating time of the image intensifier is characterized by the FWHM of the brightness curve. The time corresponding to the FWHM represents the OGW of the image intensifier. By calculating the FWHM of the curve, the OGW was determined to be 6.0 ns. This result indicates that under the condition where EGW = 12 ns, the MP-ICCD can effectively collect optical signals within a 6.0 ns time window. This OGW value can therefore be used as the MOGW for the MP-ICCD under these conditions.

### 3.2. Optimal Gain Voltage

**Figure 10:**
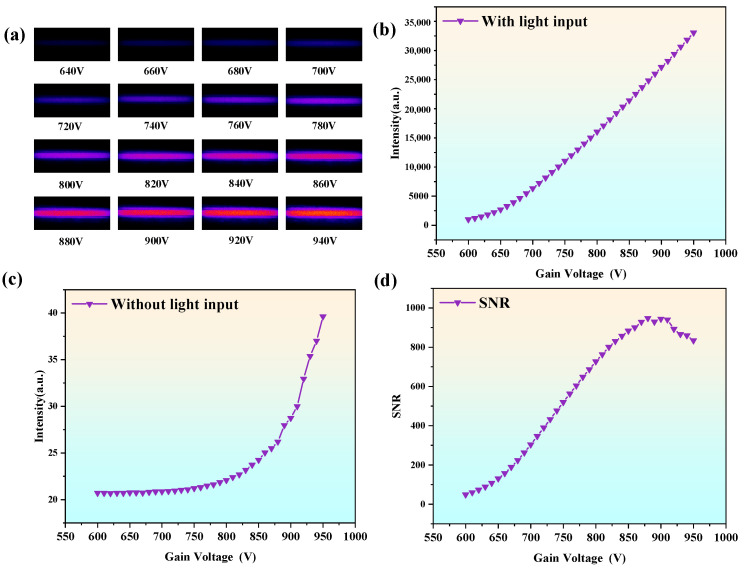
(**a**) Image showing the variation in fluorescent screen brightness with gain voltage under light input conditions. (**b**) Variation in fluorescent screen brightness with gain voltage under light input conditions. (**c**) Variation in fluorescent screen brightness with gain voltage under no light input conditions. (**d**) Variation in the SNR with gain voltage.

During testing, the gain voltage was set to range from 600 V to 950 V, with a step size of 10 V. At each gain voltage, the brightness of the phosphor screen was measured under conditions with and without light source input. Figure 10a illustrates the progressive increase in phosphor screen brightness with gain voltage (640–940 V) under light input, with brightness visualized using pseudo-color representation.

Furthermore, the average brightness value of all pixels in the bright region was calculated for all test results, as shown in Figure 10b. The phosphor screen brightness exhibited a nonlinear increase with gain voltage: a slow increase during the low-gain stage (600–750 V), followed by a rapid rise during the mid-to-high gain stage (750–950 V). Figure 10c shows the phosphor screen brightness under no light input, caused by the intrinsic noise of the image intensifier. In this case, brightness increased gradually with gain voltage, with minimal changes during the low-gain stage and significant increases at high gain levels, especially beyond 900 V, where brightness rose sharply.

Figure 10d illustrates the trend of the SNR with varying gain voltage, calculated using Equation (2). The SNR initially increases and then decreases, reaching its peak at a gain voltage of approximately 870 V. This indicates that the optical signal is most effectively amplified at this point, enabling more efficient signal detection. Comprehensive analysis reveals that the optimal operating point for the developed image intensifier is at a gain voltage of approximately 870 V. At this voltage, the phosphor screen brightness is relatively high, noise is minimal, and the SNR reaches its maximum, indicating the best overall performance.

### 3.3. Relative Resolution

**Figure 11:**
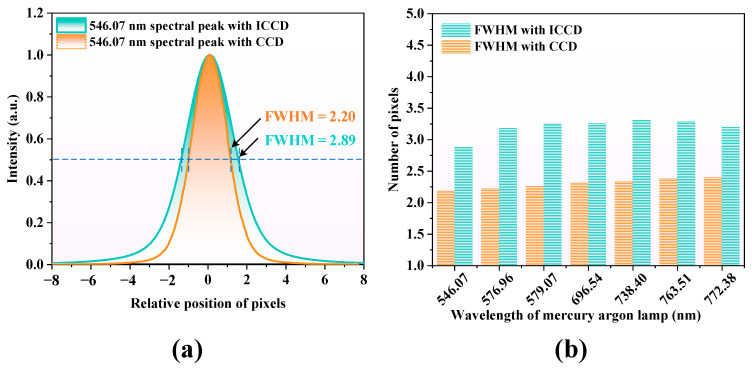
(**a**) The 546.07 nm mercury–argon lamp spectrum acquired using CCD and MP-ICCD. (**b**) Comparison of CCD and MP-ICCD resolutions.

Figure 11a shows the spectral line of the 546.07 nm wavelength light signal from a mercury–argon lamp source, captured by the spectrometer and detected using both CCD and MP-ICCD detectors. For comparison, the spectral lines obtained by the two detection methods have been normalized and aligned at their centers. The resolution of the detectors was characterized by the FWHM of the spectral lines. Analysis of the test data reveals that the FWHM of the spectral line obtained with the CCD is 2.20 pixels, while that obtained with the MP-ICCD is 2.89 pixels. The spectral line acquired with the MP-ICCD is broadened by 0.69 pixels, indicating that the resolution of the MP-ICCD at the 546.07 nm spectral line is 31% lower than that of the CCD.

Figure 11b uses a bar chart to compare the resolution differences between the MP-ICCD and CCD at seven spectral line positions ranging from 546.07 nm to 772.38 nm, further validating the disparity in their resolving capabilities. The results show that the resolution of the MP-ICCD is consistently and significantly lower than that of the CCD across all spectral line positions.

Data from the bar chart have been compiled into Table 1, revealing that the resolution differences between the CCD and MP-ICCD range from 0.69 to 0.99 pixels, with the largest difference occurring at the 579.07 nm wavelength. The MP-ICCD’s resolution degradation rate spans from 31% to 44%, reaching its maximum of 44% at 579.07 nm. Overall, the CCD demonstrates more stable resolution performance, while the MP-ICCD exhibits noticeable fluctuations in resolution at different spectral line positions.

It is important to emphasize that these test results do not reflect the inherent resolution differences at various wavelengths but rather the resolution variations at corresponding detector positions. The detected spectral lines are mapped to different spatial positions on the detector via the spectrometer, leading to resolution measurements specific to these positions.

### 3.4. Raman Spectroscopy

**Figure 12:**
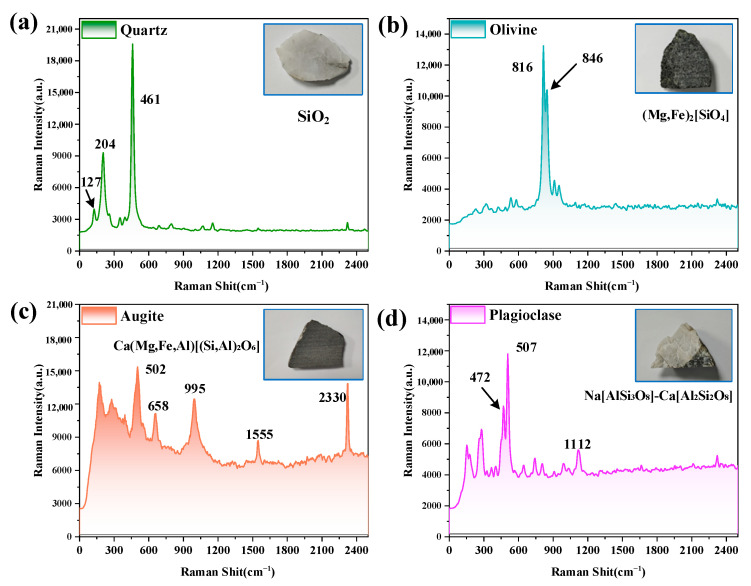
(**a**) Raman spectrum of quartz. (**b**) Raman spectrum of olivine. (**c**) Raman spectrum of augite. (**d**) Raman spectrum of plagioclase.

Figure 12 shows the Raman spectral data of four typical lunar minerals collected by the TG-LRS prototype, a remote Raman spectrometer using the MP-ICCD as the detector, under outdoor sunlight with an illuminance of 43,700 lx. The tested samples include quartz, olivine, augite, and plagioclase. Each subplot displays the Raman spectrum of the corresponding mineral, accompanied by a photograph of the mineral sample, its chemical formula, and labeled key characteristic peak positions.

During the tests, the MP-ICCD was configured with the MOGW and an optimal gain voltage of 870 V. Due to significant differences in the Raman scattering efficiency of the samples, different integration times were employed: 20 ms for quartz and olivine, 100 ms for plagioclase, and 200 ms for augite. For each sample, 100 Raman spectra were collected and averaged to enhance the signal quality.

Figure 12a shows the detected Raman spectrum of quartz (SiO_2_), with characteristic peaks at 127 cm⁻^1^, 204 cm⁻^1^, and 461 cm⁻^1^, representing typical vibrational modes of the quartz lattice. Figure 12b presents the Raman spectrum of olivine ((Mg,Fe)_2_[SiO_4_]), with prominent characteristic peaks at 816 cm⁻^1^ and 846 cm⁻^1^, reflecting stretching vibrations within its silicate structure. Figure 12c corresponds to augite (Ca(Mg,Fe,Al)[(Si,Al)_2_O_6_]), whose Raman spectrum exhibits multiple characteristic peaks, including 502 cm⁻^1^, 658 cm⁻^1^, and 995 cm⁻^1^, indicative of the complex vibrational features of its silicate framework. Figure 12d displays the Raman spectrum of plagioclase (Na[AlSi_3_O_8_]-Ca[Al_2_Si_2_O_8_]), with characteristic peaks at 472 cm⁻^1^, 507 cm⁻^1^, and 1112 cm⁻^1^, revealing vibrational properties associated with its aluminosilicate crystal structure. In Figure 12c, due to the use of a longer integration time, the Raman peaks corresponding to the stretching vibrations of oxygen and nitrogen molecules in the atmosphere are clearly observed, with their peak positions located at 1555 cm⁻^1^ and 2330 cm⁻^1^, respectively.

## 4. Discussion

In this study, we successfully developed an MP-ICCD detector tailored for lunar remote Raman spectroscopy applications. During the development process, the image intensifier was optimized to achieve high sensitivity, high resolution, and fast response. Specific optimization measures included applying anti-reflection coatings on the inner layer of the input window, adopting an integrated design of the phosphor screen and fiber optic taper, and fabricating a microstrip photocathode. Additionally, a linear CCD suitable for spectral detection applications was selected for high-precision coupling.

The results demonstrate that the MP-ICCD exhibits excellent sensitivity and optical resolution in Raman spectroscopy detection. Compared with the optical lens-coupled ICCD used in SuperCam [17,18], the MP-ICCD achieves a MOGW of 6 ns and also features a lightweight and small size, which gives it an important application advantage in lunar exploration missions.

To optimize the development of the MP-ICCD and align it with Raman spectroscopy applications, this study proposed a performance validation scheme comprising four key tests: MOGW testing, optimal gain voltage testing, relative resolution testing, and Raman signal detection. The MOGW and optimal gain voltage tests were conducted prior to coupling the MP-ICCD, while the relative resolution test was performed post-coupling. Finally, the Raman signal detection capability was validated by integrating the MP-ICCD into a prototype remote Raman spectrometer. Compared with the traditional single test methods for ICCD such as resolution, gating time, noise, etc. [34,35,36], this study proposes an integrated test method covering multiple development aspects and system levels, which achieves a systematic evaluation of the MP-ICCD.

In the MOGW test, the fastest switching speed of the developed image intensifier was measured to be 6 ns using a pulsed laser and precise timing control. The optimal gain voltage test determined the ideal gain voltage to be 870 V by adjusting the gain voltage and calculating the SNR. This finding is crucial for the rapid parameter configuration of the lunar remote Raman spectrometer during in-orbit operations. The relative resolution test, conducted by comparing the MP-ICCD with an uncoupled CCD, assessed the resolution degradation. The results indicated a resolution reduction of 31% to 44% for the MP-ICCD compared to the CCD. This highlights the importance of accounting for resolution degradation in evaluations and the need for further improvements in fabrication processes to enhance resolution. The Raman signal detection experiment provided preliminary validation of the MP-ICCD’s effectiveness in Raman spectroscopy applications.

However, this study has certain limitations. First, although this study conducted preliminary tests on the Raman spectra of typical lunar surface minerals under outdoor sunlight conditions, further research is needed to systematically investigate the performance of the MP-ICCD and the influence of various parameters on the Raman spectra. Subsequent research will be conducted on the water ice detection capability of a remote spectral detection system based on the MP-ICCD. The current tests were performed at a distance of 1.5 m, and future experiments could explore detection at greater distances to evaluate the system’s applicability. Based on the quality of the Raman spectral signals obtained in this test, the system demonstrates potential for longer-range detection. Additionally, more experiments under strong light background conditions are required to thoroughly analyze the effect of optical gate width on the signal-to-noise ratio of Raman spectra, aiming to further optimize system performance. Water ice may exist in the permanently shadowed craters at the lunar south pole. In subsequent studies, we will conduct experiments to explore the potential of detecting water ice using a remote Raman spectrometer based on the MP-ICCD. Second, although the MP-ICCD demonstrated excellent performance in this research, external factors such as radiation and temperature variations in the extreme lunar environment may impact its long-term stability and durability. Future studies are needed to further validate its performance under varying environmental conditions.

## 5. Conclusions

In summary, to meet the application requirements of lunar remote Raman spectroscopy, this study successfully developed the MP-ICCD detector through optimized image intensifier design, selection of a suitable CCD, and precise coupling. To further evaluate its performance and apply it to Raman spectrometers, a systematic performance validation scheme was proposed. Through experiments, we determined key parameters of the developed MP-ICCD, including a minimum optical gate width of 6 ns, an optimal gain voltage of 870 V, and a resolution attenuation of 31–44% relative to the CCD. These results effectively assess the comprehensive performance of the MP-ICCD.

Under outdoor sunlight conditions, the prototype remote Raman spectrometer equipped with the MP-ICCD successfully detected Raman spectra of typical lunar minerals, including quartz, olivine, pyroxene, and plagioclase, at a distance of 1.5 m. Based on the analysis and discussion of the test results, it is concluded that the MP-ICCD holds significant potential for future lunar remote Raman spectroscopy missions. It is expected to provide critical technical support for planetary science and mineralogical research.

## Figures and Tables

**Table 1:** Detailed data on resolution comparison between CCD and MP-ICCD.

No.	Mercury–Argon Lamp Spectral Lines (nm)	CCD Resolution (Pixel)	ICCD Resolution (Pixel)	Resolution Difference (Pixel)	Decay Rate
1	546.07	2.20	2.89	0.69	31%
2	576.96	2.23	3.19	0.96	43%
3	579.07	2.27	3.26	0.99	44%
4	696.54	2.32	3.27	0.95	41%
5	738.40	2.34	3.32	0.98	42%
6	763.51	2.39	3.30	0.90	38%
7	772.38	2.41	3.21	0.80	33%

## Data Availability

Data are contained within the article.
